# Gaseous 3-pentanol primes plant immunity against a bacterial speck pathogen, *Pseudomonas syringae* pv. tomato via salicylic acid and jasmonic acid-dependent signaling pathways in *Arabidopsis*

**DOI:** 10.3389/fpls.2015.00821

**Published:** 2015-10-06

**Authors:** Geun C. Song, Hye K. Choi, Choong-Min Ryu

**Affiliations:** ^1^Molecular Phytobacteriology Laboratory, Korea Research Institute of Bioscience and Biotechnology, Daejeon, South Korea; ^2^Biosystems and Bioengineering Program, University of Science and Technology, Daejeon, South Korea

**Keywords:** 3-pentanol, induced resistance, volatile organic compound, *Arabidopsis*, bacterial speck pathogen

## Abstract

3-Pentanol is an active organic compound produced by plants and is a component of emitted insect sex pheromones. A previous study reported that drench application of 3-pentanol elicited plant immunity against microbial pathogens and an insect pest in crop plants. Here, we evaluated whether 3-pentanol and the derivatives 1-pentanol and 2-pentanol induced plant systemic resistance using the *in vitro* I-plate system. Exposure of *Arabidopsis* seedlings to 10 μM and 100 nM 3-pentanol evaporate elicited an immune response to *Pseudomonas syringae* pv. tomato DC3000. We performed quantitative real-time PCR to investigate the 3-pentanol-mediated *Arabidopsis* immune responses by determining *Pathogenesis-Related* (*PR*) gene expression levels associated with defense signaling through salicylic acid (SA), jasmonic acid (JA), and ethylene signaling pathways. The results show that exposure to 3-pentanol and subsequent pathogen challenge upregulated *PDF1.2* and *PR1* expression. Selected *Arabidopsis* mutants confirmed that the 3-pentanol-mediated immune response involved SA and JA signaling pathways and the *NPR1* gene. Taken together, this study indicates that gaseous 3-pentanol triggers induced resistance in *Arabidopsis* by priming SA and JA signaling pathways. To our knowledge, this is the first report that a volatile compound of an insect sex pheromone triggers plant systemic resistance against a bacterial pathogen.

## Introduction

Plants protect themselves against diverse microbial pathogens and insects using a variety of defense mechanisms (Agrios, 2004). Among these mechanisms, induced resistance represents a unique machinery against a broad spectrum of plant pathogens (Mysore and Ryu, 2004; Eyles et al., 2010). Induced resistance is classified into two major groups: systemic acquired resistance (SAR) elicited by avirulent pathogens and induced systemic resistance (ISR) by root-associated bacteria (Ryals et al., 1996; Kloepper et al., 2004; Pieterse et al., 2009; Balmer et al., 2013). Many of the microbial determinants that elicit plant immunity have been reported (Lyon, 2007). Microbial (fungus and bacteria) cell-wall components and secreted metabolites are major groups. Among secreted metabolites, volatile organic compounds (VOCs) have been reported to induce plant immunity when applied to plants (Farag et al., 2013; Chung et al., 2015; Kanchiswamy et al., 2015a). To apply VOC under field condition, rapid evaporation of VOCs in the open field is a major challenge (Farag et al., 2013). However, recent report shows that plants can be successfully protected against plant pathogens and insect herbivores using 3-pentanol and 2-butanone (Song and Ryu, 2013; Kanchiswamy et al., 2015b). In addition, the underlying mechanism of VOC-mediated enhancement of plant immunity remains elusive (Chung et al., 2015). Particularly, the signaling pathways involved in major plant defense mechanisms, such as those that mediate the effects of salicylic acid (SA), jasmonic acid (JA), and ethylene (ET), have been intensively studied using only 2,3-butanediol and tridecane VOC within the *Arabidopsis thaliana*-*Pectobacterium carotovorum*/*Pseudomonas syringae* pathosystem (Ryu et al., 2004; Han et al., 2006; Kwon et al., 2010; Rudrappa et al., 2010; Lee et al., 2012;). Additionally, there have been no reports to our knowledge of the defense signaling induced by insect-produced VOCs.

Among insect’s produced VOCs, 3-pentanol is well-characterized with respect to its ability to induce plant immunity on pepper and cucumber plants (Song and Ryu, 2013; Choi et al., 2014). In many cases, 3-pentanol is also an important insect sex pheromone, particularly for the ambrosia beetle *M. mutatus* (Coleoptera, Curculionidae, Platypodinae), promotes the aggregation of males (Gatti Liguori et al., 2008; Funes et al., 2009, 2011), and facilitating mating behavior in several other species (Rossiter and Staddon, 1983; Bukovinszky et al., 2005; Manrique et al., 2006; Vitta and Lorenzo, 2009; Gols et al., 2011). A crucial role for 3-pentanol as an insect sex pheromone and attractant has been reported, but its function in eliciting plant defense responses against pathogens has only recently been studied (Zhuge et al., 2010; Song and Ryu, 2013).

Drench application of 3-pentanol induces an immune response in cucumber plants against angular leaf spot caused by *Pseudomonas syringae* pv. lachrymans and the sucking insect aphid (Song and Ryu, 2013). However, the molecular mechanism of 3-pentanol-mediated plant immunity is unknown. Here, we used the model plant *Arabidopsis thaliana* to investigate 3-pentanol-mediated immunity *in vitro*. We utilized the I-plate system, which is a Petri dish divided into two physically separated compartments that share the same headspace, to investigate the effects of 3-pentanol and its isoforms 1-pentanol and 2-pentanol on plant tissues and pathogen challenge. We focused on 3-pentanol activation of defense priming in plant immunity, which primes major plant defense signaling pathways involved in the plant immune response (Conrath, 2011). Defense priming is indicated by faster or stronger expression of defense-related genes by secondary biotic and abiotic stresses (Pare et al., 2005; Yang et al., 2009). We evaluated 3-pentanol-mediated defense priming in selected *Arabidopsis* mutants by performing qRT-PCR analysis of *Pathogenesis-Related* (*PR*) gene expression in SA, JA, and ET signaling pathways. Our results indicate that a volatile emission of the insect pheromone 3-pentanol elicits an induced resistance response that protects *Arabidopsis* plants against pathogen infection.

## Materials and Methods

### Disease Assay and Effect of 3-pentanol on *Arabidopsis*

**FIGURE 1:**
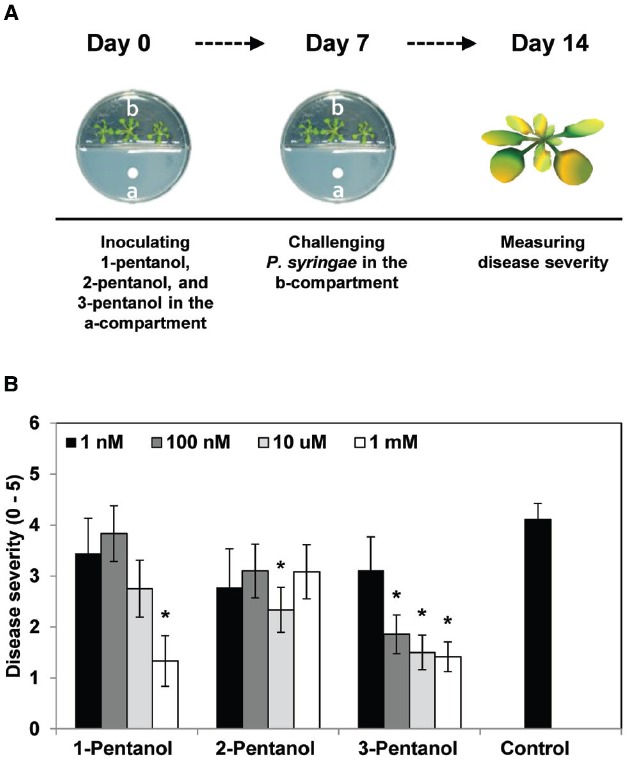
**Effects of gaseous 1-pentanol, 2-pentanol, and 3-pentanol on ***Arabidopsis*** systemic resistance. (A,B)** *Arabidopsis* seedlings were planted in an I-plate, which contains two physically separated compartments that share the same headspace. Plants are exposed to volatile 3-pentanol and its isoforms (or sterile distilled water control), which evaporates from solutions in the other compartment. All plants were drop-inoculated with *P. syringae* pv. tomato DC3000 (*Pto*) 7 days after exposure to volatiles from 1 nM, 100 nM, 10 μM, and 1 mM 1-pentanol, 2-pentanol, and 3-pentanol. Disease severity **(B)** was measured 7 days after pathogen inoculation. The asterisk in **(B)** indicates significant differences (*P* = 0.05 by LSD) between treatment and control. Error bars represent means ± SEM; *N* = 12 plants per treatment.

Plant and bacterial preparations were conducted as described previously (Ryu et al., 2003a, 2004; Lee et al., 2012). Briefly, *Arabidopsis thaliana* ecotype Columbia (Col-0) seedlings that had been lowed to germinate and grow for at least 2 days were transferred to one compartment of an I-plate (SPL Lifesciences Co., Pocheon, Gyeonggi-do, South Korea) containing 1/2 Murashige and Skoog medium supplemented with 0.6% (w/v) agar and 1.5% (w/v) sucrose. Plants were cultivated in the I-plates in a growth chamber for 14 days at 21°C under a 16 h light/8 h dark cycle before collecting samples for gene expression analysis. Bacterial pathogens were cultured overnight at 30°C in LB medium supplemented with 100 μg/ml rifampicin. *Arabidopsis thaliana* ecotype Columbia (Col-0) plants were prepared as described previously (Lee et al., 2012). The I-plate system was employed to assess induced resistance mediated by 1-pentanol, 2-pentanol, and 3-pentanol; 30 μl of 1 nM, 100 nM, 10 μM, and 1 mM of each C5 amyl alcohol (or sterile distilled water control) was added to one compartment of an I-plate (the a-compartment in Figure 1A) containing *Arabidopsis* plants in the other compartment, and the plate was tightly sealed with Parafilm. For the induced resistance assay, 2 μL of freshly prepared suspension of *P. syringae* pv. tomato DC3000 (*Pto*) in sterile distilled water [10^7^ colony-forming units (CFUs) per mL] was drop-inoculated on leaves 7 days after exposure to each C5 amyl alcohol. Sterile distilled water was mock-inoculated as a negative control. Inoculated plants were placed in a dew chamber (100% humidity) under darkness for 1 d at 25°C. Disease severity was measured 5–7 days after pathogen challenge. The disease rate (0–5) of each plant was measured by recording the percentage of total plant leaf surface showing symptoms as follows: 0 = no symptoms, 1 = mild chlorosis at the inoculated site, 2 = chlorosis covering half of the leaf, 3 = chlorosis covering the whole leaf, 4 = severe chlorosis and mild necrosis, and 5 = most severe symptoms with necrosis (Lee et al., 2012). This was designed as a completely randomized experiment with 12 replications and one plant per replication. The entire experiment was repeated three times. For long-term storage, bacterial cultures were maintained at –80°C in King’s B medium containing 20% glycerol.

### RT-PCR and qRT-PCR

For qRT-PCR analysis to investigate defense priming of signaling marker genes, Col-0 plants were exposed to 3-pentanol, subsequently challenged with pathogen, and leaf tissues were collected at 0.2 (15 min), 6, and 12 h after pathogen challenge and used for further experiments (Figure 3A). Total RNA was isolated from *Arabidopsis* leaf tissues using the TRI reagent (Molecular Research Center, USA) according to the manufacturer’s instructions. First-strand cDNA synthesis was performed with 2 μg of DNase-treated total RNA, oligo-dT primers, and Moloney murine leukemia virus reverse transcriptase (MMLV- RT, Enzynomics, Korea). PCR reactions were performed according to the manufacturer’s instructions. Expression of the candidate defense priming genes *Chinase B* (*CHIB*) for ET response, *plant defense 1.2* (*PDF1.2*) for JA response, and *Pathogenesis-related gene 1* (*PR1*) for SA response was assessed using the following primers: 5′-GCTTCAGACTACTGTGAACC-3′ (*CHIB*-F), 5′-TCCACCGTTAATGATGTTCG-3′ (*CHIB* -R); 5′-AATGAGCTCTCATGGCTAAGTTTGCTTCC-3′ (*PDF1.2*-F), 5′-AATCCATGGAATACACACGATTTAGCACC-3′ (*PDF1.2*-R); and 5′-TTCCACAACCAGGCACGAGGAG-3′ (*PR1*-F), and 5′-CCAGACAAGTCACCGCTACCC-3′ (*PR1*-R). A Chromo4 Real-Time PCR system (Bio-Rad, USA) was used for qRT-PCR. Reaction mixtures consisted of cDNA, iQTM SYBR^®^ Green Supermix (Bio-Rad), and 10 pM of each primer. Thermocycler parameters were as follows: initial polymerase activation, 10 min at 95°C; then 40 cycles of 30 s at 95°C, 60 s at 55°C, and 30 s at 72°C. Conditions were determined by comparing threshold values in a series of dilutions of the RT product, followed by a non-RT template control and a non-template control for each primer pair. Relative RNA levels were calibrated and normalized to the level of *AtAct2* mRNA.

### Induced Resistance in *Arabidopsis* Mutants *npr1*, *sid2*, *jar1-1*, and *etr1-3* and Transgenic NahG Plants

To test whether 3-pentanol elicits induced resistance via the JA, SA, or ET pathway, *Pto*-induced disease development was assessed in wild-type Col-0 seedlings and the following mutants: *jar1-1* for JA signaling; *npr1*, NahG, and *sid2* for SA signaling; and *etr1-3* for ET signaling. The experimental protocols were essentially the same as those described previously (Ryu et al., 2003b). Briefly, all mutant and transgenic lines were derived from the parental *A. thaliana* ecotype Columbia (Col-0), which was obtained from the Ohio State University Stock Center, Columbus, OH, USA. The *Arabidopsis* seeds were surface-sterilized with 6% sodium hypochlorite, washed four times with SDW, and maintained at 4°C for 2 d to enhance germination. Seedlings preparation and growth condition were same as describe previously (Ryu et al., 2004). Bacterial pathogens were cultured overnight at 30°C in King’s B medium supplemented with 100 μg/ml rifampicin (Ryu et al., 2004; Lee et al., 2012). The disease symptoms were photographed 7 days after pathogen challenge. The disease rate (0–5) of each plant was measured by recording the percentage of total plant leaf surface showing symptoms as described above (Lee et al., 2012).The experimental protocol was designed to ensure complete randomization with 12 replications and one plant per replication. The entire experiment was repeated twice.

### Statistical Analysis

Analysis of variance for experimental datasets was performed using JMP software version 5.0 (SAS Institute Inc., Cary, NC, USA). Significant treatment effects were determined by the magnitude of the *F* value (*P* = 0.05). When a significant *F* test was obtained, separation of means was accomplished by Fisher’s protected LSD at *P* = 0.05.

## Results

### Gaseous 3-pentanol Triggers Induced Systemic Resistance in *Arabidopsis*

**FIGURE 2:**
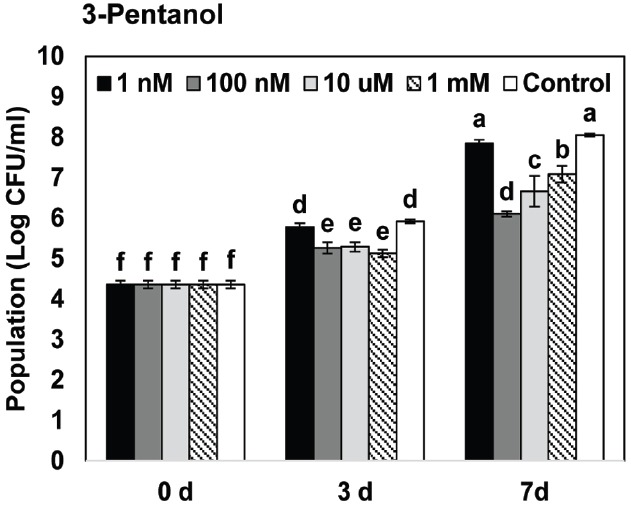
**Effects of gaseous 3-pentanol-elicited induced resistance on bacterial growth of ***P. syringae*** pv. tomato DC3000 on ***Arabidopsis.***** *Arabidopsis* seedlings were planted in an I-plate, which contains two physically separated compartments that share the same headspace. Plants are exposed to volatile 3-pentanol evaporating from the solution in the other compartment. All plants were drop-inoculated with *P. syringae* pv. tomato DC3000 (*Pto*) 7 days after exposure to volatiles from 1 nM, 100 nM, 10 μM, and 1 mM 3-pentanol. The bacterial cell count was measured 7 days after pathogen inoculation. Different letters indicate significant differences between treatments (*P* = 0.05 by LSD). Error bars represent means ± SEM; *N* = 12 plants per treatment.

We first evaluated the effect of gaseous 3-pentanol on induced resistance against *Pto* DC3000 (Figure 1A). Disease severity after exposure to volatile emission from 1 nM, 100 nM, 10 μM, and 1 mM 3-pentanol was 3.1(error range: 0.6549), 1.9 (0.3758), 1.5 (0.3371), and 1.4 (0.2875), respectively, whereas it was 4.1 (0.3093) for the mock-inoculated water control (Figure 1B). Exposure to volatile emission from 1 mM 1-pentanol and 10 μM 2-pentanol significantly reduced disease severity compared to control. These experiments show that 3-pentanol is more effective than other isoforms to induce resistance against *Pto* DC3000 and a concentration of 100 nM was sufficient to significantly reduce disease severity (Figure 1B). The number of bacterial cells in leaf collected 3 and 7 days after inoculation was reduced significantly in plants exposed to 100 nM, 10 μM, and 1 mM 3-pentanol, whereas bacterial growth was not significantly different in plants exposed to 1 nM 3-pentanol and control plants (Figure 2. The results show that 10 and 100 nM 3-pentanol reduced bacterial cell counts by 100- and 25-fold, respectively, compared with mock-inoculated control. Therefore, we chose 10 μM and 100 nM 3-pentanol for further experiments. No direct inhibition was detected between different concentrations of 3-pentanol and *Pto* DC3000 indicating that the population reduction was caused by elicitation of induced resistance (data not shown).

### 3-pentanol-Mediated Induced Resistance Involves SA and JA Signaling Pathways

**FIGURE 3:**
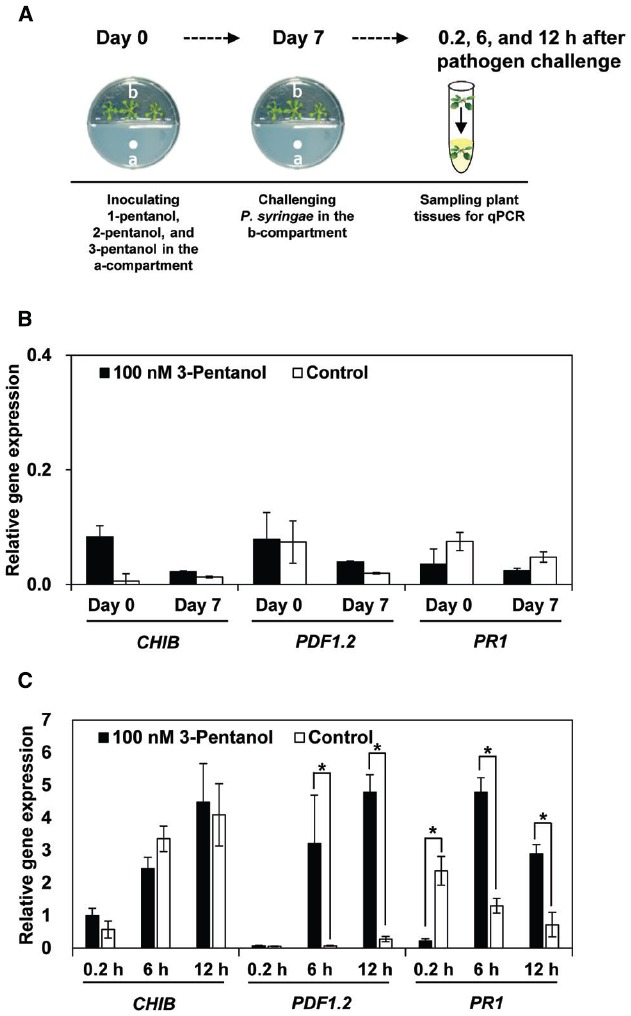
**Expression marker genes for defense priming of SA, JA, and ET signaling pathways in ***Arabidopsis*** Col-0 plants treated with volatile 3-pentanol (100 nM) and subsequently challenged with ***P. syringae*** pv. tomato DC3000 (***Pto***) (A).** Expression of *PR1*, *PDF1.2*, and *CHIB* was analyzed with qRT-PCR at 0 and 7 days after volatile 3-pentanol treatment **(B)** and then at 0, 6, and 12 h after *Pto* inoculation **(C)**. The housekeeping gene, *AtActin*, was used to indicate equal loading. Asterisks indicate significant differences among treatments (*P* = 0.05 by LSD). Error bars represent means ± SEM; *N* = 12 plants per treatment.

We evaluated the expression of defense-related genes in response to 3-pentanol and subsequent challenge with *Pto* DC3000 using Quantitative RT-PCR (qRT-PCR; Figure 3A). We first investigated the direct effect of 3-pentanol on the defense-related genes of *Arabidopsis*, including *PR1* (SA response), *PDF1.2* (JA response), and *CHIB* (ET response) that evaluated previously (Lee et al., 2012). The transcriptional level of the three signaling marker genes did not differ greatly from the control at day 0 and 7 after exposure to 3-pentanol before pathogen challenge (Figure 3B). Exposure to gaseous 3-pentanol and subsequent pathogen challenge caused a 32- and 16-fold upregulation of *PDF1.2* (JA signaling) and a 3.7- and 4.1-fold upregulation of *PR1* (SA signaling) transcriptional level, respectively, compared with that of sterile distilled water control at 6 and 12 h post inoculation (hpi). By contrast, there was no significant difference in *CHIB* (ET signaling) transcriptional level at 0.2, 6, and 12 hpi in plants exposed to gaseous 3-pentanol or sterile distilled water control and subsequently challenged with pathogen. These results suggest that 3-pentanol treatment primes the JA and SA signaling pathways (Figure 3C). Interestingly, significant upregulation of *PR1* at 0.2 hpi was detected in control plant when compared to that of gaseous 3-pentanol treated plants (Figure 3C).

**FIGURE 4:**
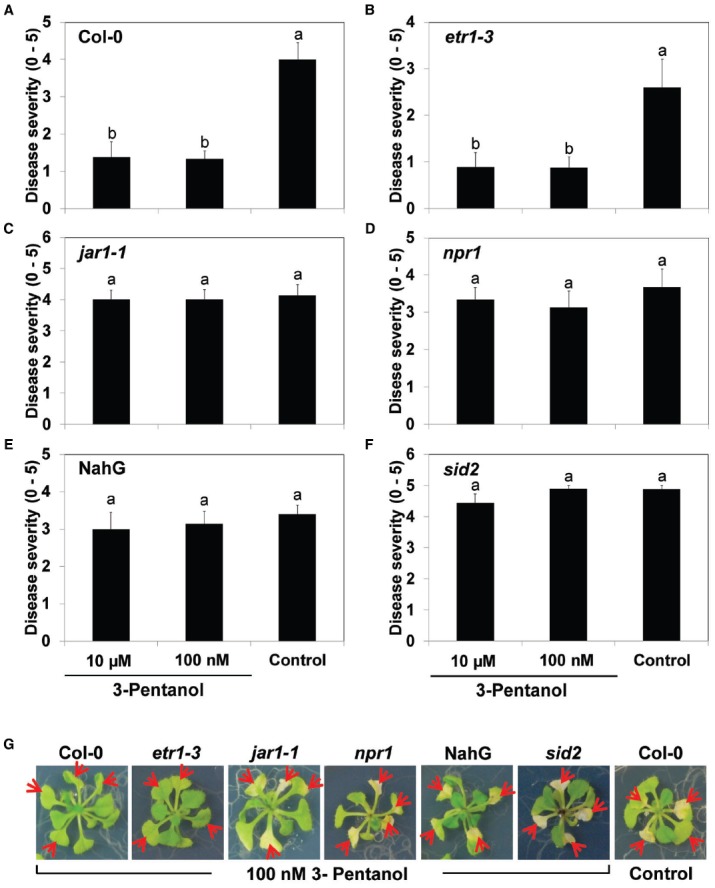
**Volatile 3-pentanol-mediated induced resistance in ***Arabidopsis*** mutants against ***Pto***.** *Arabidopsis* Col-0 seedlings **(A)**, *etr1-3* **(B)**, *jar1-1* **(C)**, *npr1* **(D)**, NahG **(E)**, and *sid2* **(F)** were exposed to 100 nM and 10 μM 3-pentanol evaporate or sterile distilled water control. After 5 days, all plants were drop-inoculated with *Pto*. Disease severity was measured 7 days after pathogen inoculation. Numbers represent means of 12 replications per treatment, with one seedling per replication. Different letters in **(A)** to **(F)** indicate significant differences between treatments (*P* = 0.05 by LSD). Error bars represent means ± SEM. **(G)** Plants were photographed 7 days after pathogen challenge.

Next, we used the I-plate system to test the effects of 100 nM and 10 μM 3-pentanol evaporate on plant defense pathway signaling in five *Arabidopsis* mutants [*npr1* (SA signaling), NahG (SA degradation), *sid2* (SA synthesis), *jar1-1* (JA-resistant), and *etr1-3* (ET receptor mutant)] challenged with *Pto* DC3000 (Lee et al., 2012). The SA signaling-related mutants *npr1*, NahG, and *sid2*, and the JA-resistant mutant *jar1-1* displayed severe disease symptoms (Figures 4C–G), whereas Col-0 and *etr1-3* consistently displayed induced resistance (Figures 4A,B, G). We obtained similar results from three independent experiments.

### Discussion

Our previous results showed that drench application of 3-pentanol protected cucumber plants against bacterial pathogens and insects in an open-field experiment (Song and Ryu, 2013). In the current study, we took a step further and evaluated the molecular mechanisms behind 1-pentanol, 2-pentanol, and 3-pentanol-mediated plant systemic resistance. We also investigated which plant defense signaling pathways may be primed by 3-pentanol-mediated induced resistance. This study shows that exposure to gaseous 3-pentanol upregulates the expression of marker genes *PDF1.2* and *PR1*, thereby indicating that 3-pentanol primes JA and SA defense signaling pathways.

Plant-associated bacteria including mainly *Bacillus* spp. and *Pseudomonas* spp. release 10–40 VOCs into the headspace above colonies grown on complex medium (Farag et al., 2013). *In planta*, 2,3-butanediol or acetoin were identified as bacterial VOCs responsible for reducing soft-rot symptoms caused by *Pectobacterium carotovorum* subsp. carotovorum in *Arabidopsis*, anthracnose caused by *Colletotrichum orbiculare* in *Nicotiana benthamiana*, and the fungal pathogens, *Microdochium nivale*, *Rhizoctonia solani*, or *Sclerotinia homoeocarpa* in *Agrostis stolonifera*, suggesting the possible use of bacterial VOCs as environmentally sound biochemical agents for agricultural applications (Ryu et al., 2004; Chung et al., 2015). However, there are several disadvantages to the use of bacterial VOCs in agricultural fields: (1) rapid evaporation rate, (2) inconsistencies between *in vitro* effects (e.g., I-plate) of VOCs and effects observed in open-field experiments, and (3) unstable effectiveness of target VOCs. To overcome these problems, we attempted to identify more effective bacterial volatiles and their derivatives that increased plant resistance. Previous studies on bacterial volatiles as chemical triggers of systemic resistance were carried out in cucumber (Song and Ryu, 2013). We initially identified 1-pentanol from a headspace analysis of bacterial colonies, and then assessed the derivative 3-pentanol as a trigger for systemic resistance in pepper plants cultivated in a greenhouse (data not shown). Unexpectedly, 3-pentanol had greater effect on induced resistance in pepper than 1-pentanol. We then evaluated the effectiveness of 3-pentanol in an open-field experiment. Drench application of 1 mM 3-pentanol into the soil conferred plant protection against bacterial spot caused by *Xanthomonas axonopodis* pv. vesicatoria (Choi et al., 2014). In the greenhouse and open-field experiments, we cannot rule out an effect of 3-pentanol as a VOC that triggers the induction of systemic resistance due to volatilization (evaporation) of 3-pentanol after drench application.

We tested the effect of gaseous 3-pentanol on induced resistance in *Arabidopsis* seedlings grown in I-plates; the results indicate that symptom development was significantly suppressed (Figures 1 and 2). Drench application of 3-pentanol to *Arabidopsis* seedlings grown under *in vitro* conditions triggered induced resistance (data not shown). Disease development was not significantly different between the two application protocols (exposure to gaseous 3-pentanol or drench application to the roots), indicating that volatile 3-pentanol may be the main agent triggering ISR in drench application experiments. The previous study showed a significant behavioral response of the ambrosia beetle, *M. mutatus*, to 10 μg of 3-pentanol (= 100 nM 3-pentanol) (Manrique et al., 2006). The current study evaluated different 3-pentanol doses and clearly demonstrated biological relevance for triggering plant defense responses (Figures 1B,C). 3-pentanol did not show direct growth inhibition of *Pto* (data not shown). Furthermore, 3-pentanol volatile applications displayed no significant increase of *CHIB*, *PDF1.2*, and *PR1* expression levels (Figure 3B). Alternatively, 3-pentanol considerably primed the elicitation of transcriptional levels of *PDF1.2* and *PR1* when compared to controls after the pathogen challenge (Figure 3C). Thus the results clearly indicated that gaseous 3-pentanol mediate defense priming of SA (*PR1* marker gene upregulation) and JA (*PDF1.2* marker gene upregulation) signaling pathways (Figures 3B,C).

An unexpected increase in the level of *PR1* gene transcription at 0.2 h in the control plant (Figure 3C) can be speculated as wound-mediated defense priming, that is suppressed by *Pto* at 6 and 12 h (Figure 3C). Nevertheless, the detailed underlying mechanism remains to be understood.

This result is in agreement with a previous report. Drench application of 100 nM 3-pentanol on pepper seedlings primed the transcriptional level of pepper defense genes including *CaPR1* and *CaPR2* for SA signaling, *CaPIN2* for JA signaling, and *CaPR4* and *CaGLP1* for ET signaling at 3 and 6 h after pathogen challenge (Choi et al., 2014). In cucumber, drench application of 1 mM 3-pentanol upregulated *CsLOX1* at 6 h after pathogen challenge, indicating that the oxylipin pathway was triggered to recruit the natural enemy of aphids (Song and Ryu, 2013).

## Conclusion

In conclusion, this study shows that a volatile 3-pentanol can trigger plant systemic resistance against *Pto* DC3000. Our results suggest that insect pheromones may be a rich source of chemical triggers that protect plants by enhancing plant immunity.

### Conflict of Interest Statement

The authors declare that the research was conducted in the absence of any commercial or financial relationships that could be construed as a potential conflict of interest.

## Abbreviations

Col-0: *Arabidopsis thaliana* ecotype Columbia
ET: ethylene
JA: jasmonic acid
*PR*: *Pathogenesis-Related* genes
*Pto*: *Pseudomonas syringae* pv. tomato DC3000
qRT-PCR: quantitative RT-PCR
SA: salicylic acid
VOC: volatile organic compound.
